# Some Brief Notes on Medicines Used in Veterinary Practice

**Published:** 1889-01

**Authors:** Kenelm Winslow

**Affiliations:** Instructor in Materia Medica and Botany, Veterinary Department, Harvard University


### ART. III.—SOME BRIEF NOTES ON MEDICINES USED IN VETERINARY PRACTICE.

BY KENELM WINSLOW, B. A. S., M. D. V.,
*Instructor in Materia Medica and Botany, Veterinary Department, Harvard University.*

### ZINCI OXIDUM.

Oxide of Zinc is commonly regarded in both human and veterinary practice as being an entirely innocuous agent, but on applying the officinal ointment (1-4) generously over the abdomen of a fox terrier dog suffering from sub-acute eczema, the most violent paroxysms of pain manifested themselves after the second application, resembling a violent colic, with some symptoms of delirium and acute coryza. The dog was supposed to be in a "fit," and was therefore immediately plunged into hot water. When seen by me there was considerable swelling and congestion of the skin and subcutaneous tissue, and after prescribing carron oil and following it up with a dressing of starch and subnitrate of bismuth, recovery occurred shortly and the eczema disappeared in like manner within a week or ten days. The ointment was found on analysis to be uncontaminated with impurities and the result is to be ascribed to an idiosyncrasy on the part of the patient. As it appears on investigation that such an unfavorable action rarely occurs in both human and veterinary experience, it would seem open to question whether it is desirable to apply zinc ointment over a large surface of broken skin without experimenting first in a small way upon the patient. For although a cure is the wished-for result, death would be an unpleasant and complicating feature in its attainment. As once remarked by a well known therapeutist, when contrasting the comparative value of ether and chloroform, and having praised the many superior qualities of the latter, said in conclusion "ether is more expensive but less so than a funeral." Terse but characteristic of the man, Professor Robert T. Edes.

CALABAR BEAN—PHYSOSTIGMA AND ITS ALKALOID PHYSOSTIGMINE.

With a fluid extract my experience began in such an interesting way that it has led to further trials. An aged gelding used in the trucking business, appropriately, if not elegantly, described in veterinary parlance as a "screw," after suffering for a week with impaction of the colon, without passing any dung, and being nourished with oil and stimulants, was given ten drops of a fluid extract of physostigma. Death occurred almost instantaneously with paraplegia, profuse sweating and muscular tremors, but without purging. The extract had been used in doses of from ten to twelve gtt. previously without bad effects, and it might be said, perhaps, with equal truth, without any good ones. The subject was of course an extremely unfavorable one.

Experimentally three gr. of the sulphate of physostigmine were injected within the jugular of a bay gelding placed on the dissecting table. He was aged, but of a good constitution. No marked symptoms except muscular twitchings, especially of the neck, diasphrexis and stertorous breathing occurred. After twenty-five minutes had elapsed three gr. more were given in the same way. The respiration became very labored, muscular tremors general, sweating most profuse, salivation, and in about half an hour after this last administration the sphincter relaxed and some dung with considerable flatus was expelled.

The eye was amaurotic and protruding, and it appeared that dissolution was at hand, but, as after three hours the subject seemed to be recovering, he was killed for dissection. Here it was apparent that motor paralysis was complete before evacuation of the bowels occurred and was a sign of approaching death. The one-twentieth (1-20) of a grain of alkaloidae sulphate given sub. cu. to a pointer dog, had a moderate cathartic effect within twenty minutes, without any appreciable symptoms, except muscular tremors.

But in the somewhat extensive clinical experience at the Harvard Veterinary Hospital its therapeutic value seems to be still a matter of considerable doubt, and two grains of the alkaloid have more often failed to produce any pronounced beneficial effect, but alarming symptoms have sometimes occurred.

In one case it was deemed necessary to give atropine as an antidote. Colic, impaction and constipation patients have been those commonly treated with thus drug, although azoturia and some other have been experimented with.

In human practice physostigma given internally is regarded more as a synergist than as an agent to rely on entirely in the production of catharsis.

For it seems to me that it might be justly argued whether it is advisable to administer Calabar bean in the ordinary way, *i. e.*, in such large doses for its immediate purging action. What is wished for is a tetanic state of the muscular coat of the intestines, corresponding to that observed in the external muscles, to increasing peristalsis together with augmented secretions. But this does not occur at all, or else lapses so quickly into the secondary action with large dozes, viz.: complete intestinal paralysis and relaxation as in the case above cited, that it becomes doubtful whether the therapeutic result is not as much to be dreaded as the existing disease, assuredly when the patient succumbs instanter, as in my first case, it is difficult to make the owners take a cheerful and scientific view of the experiment, they are as a rule unfortunately *too* ignorant. It will be difficult, naturally, to persuade those who have repeatedly used physostigma with apparent success, and I presume there are such to be found, that such a result is a mere hallucination, but often catharsis could naturally occur in such cases as are most frequently treated with it. Such objections are, of course, always in order as applied to clinical reasoning. There seems to me no good reason why the name of "ordeal" bean originally applied to the drug might not well be retained in view of the action of Calabar bean. It was formerly employed to test the criminality of the native Africans, and if they survived the test they were regarded as innocent on the principle of the "survival of the fittest." So in the present therapeutic application, it appears as if physostigma were used to try the strength of constitutions, as the subjects often have to pass through a fearful ordeal, and it not rarely happens that they do not succeed in passing through it, to speak rather parodoxically.

### CANNABIS INDICA—INDIAN HEMP.

The study of this drug possesses a peculiar fascination for one who has investigated its effects upon the human being.

The curious and diverse hallucinations resulting from it being especially interesting, so much so that experiments with Indian hemp are almost daily occurrences with students.

It may not be generally known that "Hashish," a preparation of Cannabis Indica, consisting of the leaves and stems with preserved fruits and aromatics, possess a singularly interesting etymological significance in our language.

The name assassin being derived from the Indian "Haschaschins" or assassins, the victims of the drug who were often led into committing violence when under the influence of hemp.

Cannabis Indica has been recommended as a substitute for opium, being devoid of some of the ill after effects of the latter drug. It may be of use as an antispasmodic and hypnotic, but when a rapid analgesiac effect is desired it seems, from the very meagre trial I have given it, to be inferior. A strong bay gelding was given an half ounce (℥ss.) of the extr. Cannabis Indica in a ball, at about 10 A. M., one morning, with directions to give two drachms more (ʒii.) later if no symptoms were developed. Very slight drowsiness and slight dilatation of the pupil was noticed, and in about two hours from the first administration he received an half ounce more (℥ss.) by accident. From this time drowsiness deepened into stupor and then into complete narcosis.

At about 3.30 P. M., amputation of the penis was performed without apparently causing the patient the slightest annoyance.

The stupor became so intense that actual paralysis occurred, and the patient fell down in a heap and had to be propped up in slings during the evening. This condition of things lasted for nearly three days, during which time the patient presented all the appearance of "dead drunkenness," as seen in his superior being and master. Ammoniacal stimulants were employed to hasten recovery. The dose was excessive, but the action was correspondingly tardy. For this reason it would seem that where pain is to be relieved opium or morphine are better agents, acting more quickly and being eliminated likewise.

If the effect which cannabis indian has on the human brain, to make the shortest unit of time seem interminable, was felt by this unfortunate animal, his existence during those three days must have, to him, included geological ages.

